# Supplementary material for: Generalizability, Robustness and Replicability When Evaluating Wellbeing of Laboratory Mice with Various Methods
Source: Animals (Basel). 2022 Oct 25;12(21):2927. doi: 10.3390/ani12212927 (PMC9654551; doi:10.3390/ani12212927)
Supplement: Supplementary file 1 [file animals-12-02927-s001.zip › animals-1945433-supplementary.pdf]

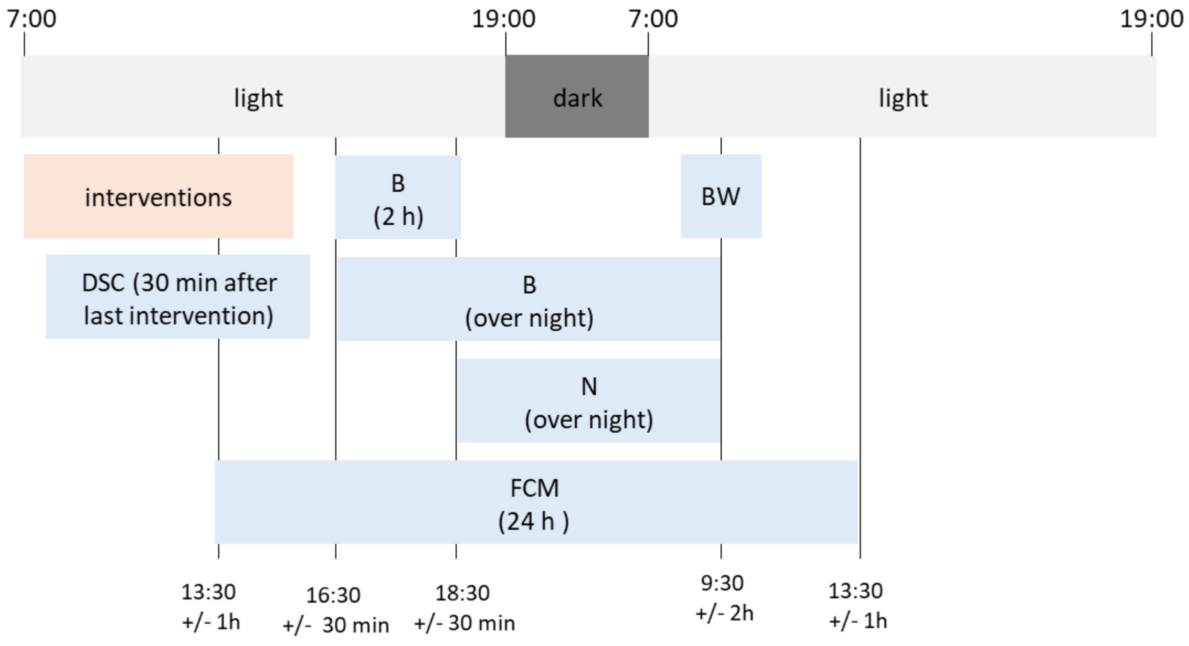

Figure S1. Timeframe of measuring animal distress. The distress score (DCS) was assessed 30 minutes after the last intervention (e.g. after the third cerulein injection, CCl4 injection, application of chemotherapy or after anesthesia plus surgical intervention). The burrowing assay (B) was started at 16:30 and evaluated after 2 hours and on the following morning. Nesting assay was started at 18:30 and also evaluated on the following morning. The mice were put in a new cage at 13:30 so that 24 hours later the fresh feces could be collected in order to assess fecal corticosterone metabolites (FCM). The body weight was measured on the following morning, because any reduction of the body weight in response to distress needs sufficient time.

A

Pre (healthy)	Post (diseased)
1.31	-1.67
1.12	-1.76
0.77	-1.88
0.67	-2.23
0.45	-2.61
0.11	-3.25
-0.65	-3.34
-0.67	-4.42
-1.21	-4.51
-1.41	-4.62

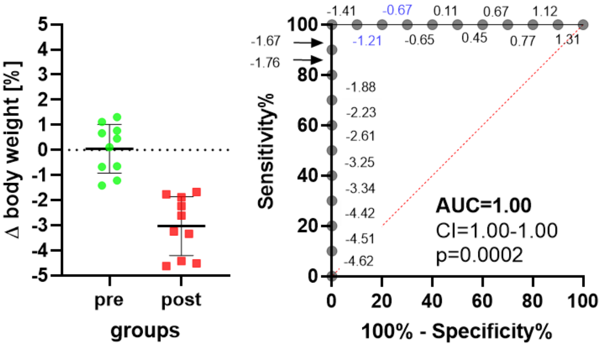

B

Pre (healthy)	Post (diseased)
1.31	-1.15
1.12	-1.25
0.77	-1.88
0.67	-2.23
0.45	-2.61
0.11	-3.25
-0.65	-3.34
-0.67	-4.42
-1.21	-4.51
-1.41	-4.62

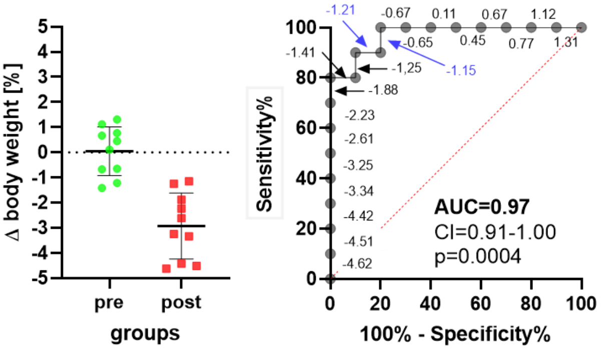

C

Pre (healthy)	Post (diseased)
1,31	1,31
1,12	1,12
0,77	0,77
0,67	0,67
0,45	0,45
0,11	0,11
-0,65	-0,65
-0,67	-0,67
-1,21	-1,21
-1,41	-1,41

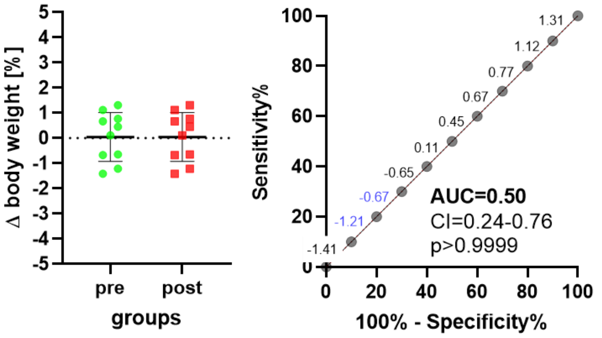

Figure S2. Example of ROC curve analysis. Fictional data before induction of a disease (pre = healthy) and after induction of a disease (post = diseased) are presented as data tables, dot blots and ROC curves in order to demonstrate how data are used to graph a ROC curve. In case all diseased animals loose more weight than healthy animals, measuring the body weight is the perfect method to differentiate between healthy and diseased animals (A). The sensitivity and specificity of various cut offs are presented in a ROC curve. For example, a cut-off between -1.21% and -0.67% body weight change allows to diagnose diseased animals with a sensitivity of 100% (because all diseased animals are diagnosed to be diseased) and a specificity of 80% (because only 8 out of 10 healthy animals are diagnosed to be healthy). Plotting sensitivity and 1-specificity for all other cut-offs results in a ROC curve with an area under the curve (AUC) of 1.00, a 95% confidence interval (CI) of 1.00-1.00 and a p-value of 0.0002. In case most diseased animals lose more weight than healthy animals, measuring the body weight is a good method to differentiate between healthy and diseased animals (B). For example, a cut-off between -1.21% and -1.15% body weight change allows to diagnose diseased animals with a sensitivity of 90% (because 9 out of 10 diseased animals are diagnosed to be diseased) and a specificity of 80% (because only 8 out of 10 healthy animals are diagnosed to be healthy). Plotting sensitivity and 1-specificity for all other cut-offs results in a ROC curve with an AUC of 0.97, a CI of 0.91-1.00 and a p-value of 0.0004. In case diseased animals have similar body weight change than healthy animals, this method cannot differentiate well between healthy and diseased animals (C). For example, a cut-off of between -1.21% and -0.67% body weight change allows to diagnose diseased animals with a sensitivity of 20% (because 2 out of 10 diseased animals are diagnosed to be diseased) and a specificity of 80% (because 8 out of 10 healthy animals are diagnosed to be healthy). A ROC curve based on all cut-offs has an AUC of 0.50, a CI of 0.24-0.76 and a p-value of > 0.9999.

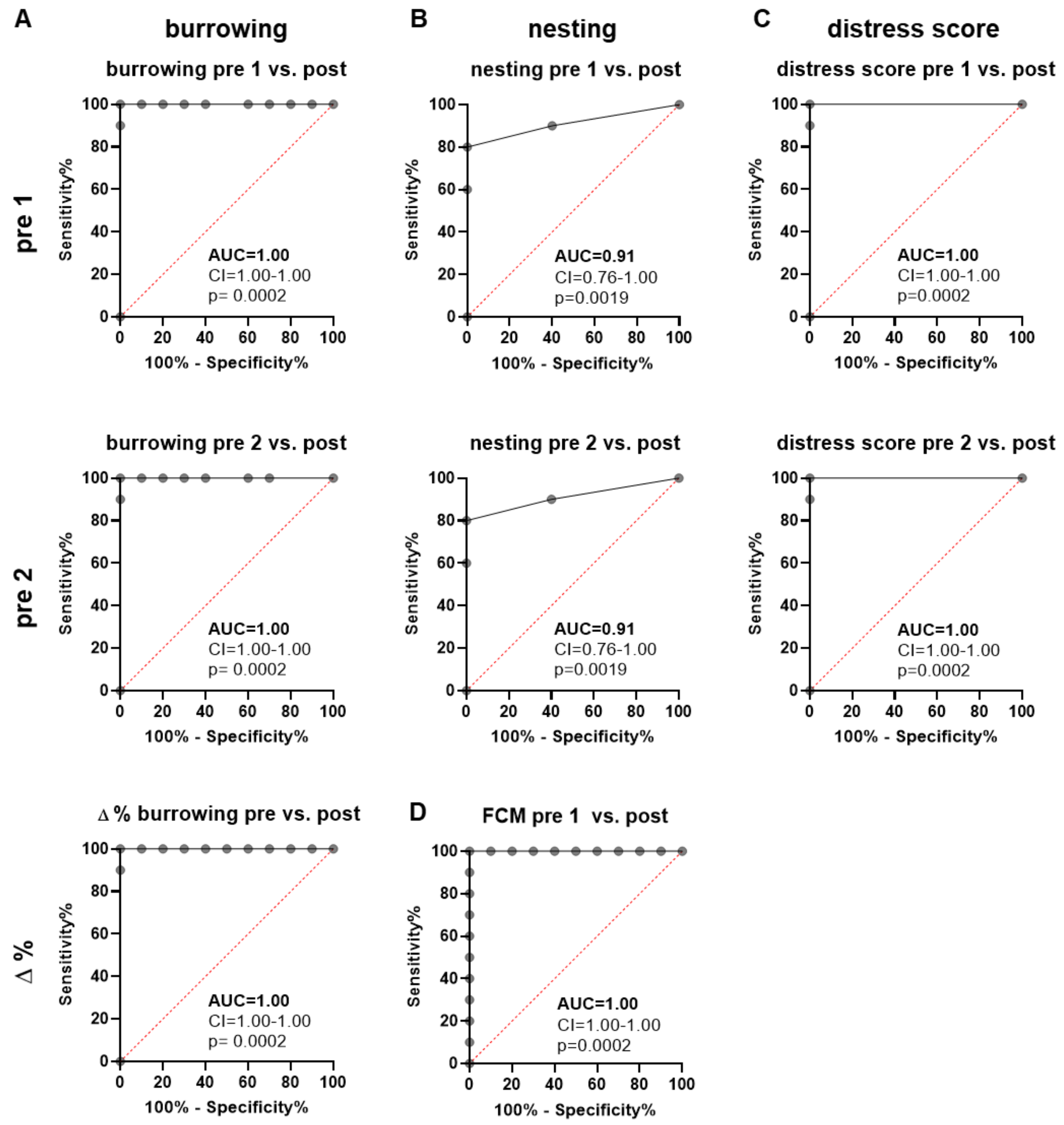

Figure S3. Presentation of classifier performance of various methods when differentiating between animals before and after implanting a telemetric transmitter. ROC curves describe changes in burrowing activity (A), nesting behavior (B) a distress score (C) or fecal corticosterone metabolites concentration (D). Burrowing activity, nesting behavior and distress score after transmitter implantation are compared to a timepoint, pre 1, or to another time point, pre 2, before implantation. In addition, the percentage in burrowing activity between the two days before transmitter implantation, is compared to the percentage in body weight change between the postoperative day and pre 1. The classifier performance of each method in differentiating between animals before and after implanting a transmitter was characterized by the area under the curve (AUC) the confidence interval (CI) and the p value indicating how significant the difference was to the reference line (red dotted line). n = 10 mice.

Table S1. Supplemental information to ROC curve analysis of figure 2. Analysis of various methods such as body weight (BW), burrowing activity (B), nesting behavior (N), a distress score (DSC) or fecal corticosterone metabolites concentration (FCM), when differentiating between animals before and after three surgical interventions. The table presents the number of animals (n) analyzed, the area under the curve (AUC), the confidence interval (CI), and the p value describing the significant difference to a reference line, which indicates no discriminative power and thus has an AUC of 0.5.

	BW	B	N	DSC	FCM
transm. impl. pre 1	n = 10<br>AUC = 0.8950<br>CI = 0.7462-1.000<br>p = 0.0028	n = 10<br>AUC = 1.000<br>CI = 1.000-1.000<br>p = 0.0002	n = 10<br>AUC = 0.9100<br>CI =0.7641-1.000<br>p = 0.0019	n = 10<br>AUC = 1.000<br>CI = 1.000-1.000<br>p = 0.0002	n = 10<br>AUC = 1.000<br>CI = 1.000-1.000<br>p = 0.0002
transm. impl. pre 2	n = 10<br>AUC = 8.8900<br>CI =0.7337-1.000<br>p = 0.0032	n = 10<br>AUC = 1.000<br>CI = 1.000-1.000<br>p = 0.0002	n = 10<br>AUC = 0.9100<br>CI = 0.7641-1.000<br>p = 0.0019	n = 10<br>AUC =1.000<br>CI = 1.000-1.000<br>p = 0.0002	not done
transm. impl. Δ %	n = 10<br>AUC = 1.000<br>CI = 1.000-1.000<br>p = 0.0002	n = 10<br>AUC = 1.000<br>CI = 1.000-1.000<br>p = 0.0002	not done	not done	not done
cell. inject. pre 1	n = 14<br>AUC = 0.5816<br>CI = 0.3650-0.7983<br>p = 0.4622	n = 14<br>AUC = 0.9184<br>CI = 0.8214-1.000<br>p = 0.0002	n = 14<br>AUC = 0.5179<br>CI = 0.2943-0.7414<br>p = 0.8722	n = 14<br>AUC = 0.500<br>CI = 0.2824-0.7176<br>p > 0.9999	n = 14<br>AUC = 0.8571<br>CI = 0.7092-1.000<br>p =0.0013
cell. inject. pre 2	n = 14<br>AUC = 0.5714<br>CI = 0.3559-0.7870<br>p =0.5201	n = 14<br>AUC = 0.8776<br>CI = 0.7405-1.000<br>p = 0.0007	n = 14<br>AUC = 0.6071<br>CI = 0.3928-0.8215<br>p = 0.3346	n = 14<br>AUC = 0.500<br>CI =0.2824-0.7176<br>p > 0.9999	not done
cell. inject. Δ %	n = 14<br>AUC = 0.5689<br>CI = 0.3510-0.7868<br>p = 0.5351	n = 14<br>AUC = 0.9184<br>CI = 0.8047-1.000<br>p = 0.0002	not done	not done	not done
BDL pre 1	n = 16<br>AUC = 0.5449<br>CI = 0.3417-0.7282<br>p = 0.6647	n = 16<br>AUC = 0.9434<br>CI = 0.8560-1.000<br>p < 0.0001	n = 14<br>AUC = 0.9898<br>CI = 0.645-1.000<br>p < 0.0001	n = 16<br>AUC = 0.9375<br>CI = 0.8392-1.000<br>p < 0.0001	not done
BDL pre 2	n = 16<br>AUC = 0.5957<br>CI = 0.3963-0.7951<br>p = 0.3558	n = 16<br>AUC = 0.8906<br>CI = 0.7755-1.000<br>p = 0.0002	n = 14<br>AUC = 0.9949<br>CI = 0.9784-1.000<br>p < 0.0001	n = 16<br>AUC = 0.9375<br>CI = 0.8392-1.000<br>p < 0.0001	not done
BDL Δ %	n = 16<br>AUC = 0.9141<br>CI = 0.8196-1.000<br>p < 0.0001	n = 16<br>AUC = 0.8867<br>CI = 0.7728-1.000<br>p = 0.0002	not done	not done	not done

Table S2. Supplemental information to ROC curve analysis of figure 3. Analysis of various methods such as percentage of body weight change (%BW), burrowing activity (B), nesting behavior (N), a distress score (DSC) or fecal corticosterone metabolites concentration (FCM), when differentiating between animals before and after two surgical interventions. The table presents the number of animals (n) analyzed, the area under the curve (AUC), the confidence interval (CI), and the p value describing the significant difference to a reference line, which indicates no discriminative power and thus has an AUC of 0.5.

	%BW	B	N	DSC	FCM
cell inject. (A)	n = 7<br>AUC = 0.6122<br>CI = 0.2928-0.9317<br>p = 0.4822	n = 7<br>AUC = 0.8571<br>CI = 0.6577-1.000<br>p = 0.0253	n = 7<br>AUC = 0.5612<br>CI =0,2448-0.8776<br>p = 0.7015	n = 7<br>AUC = 0.500<br>CI = 0.1870-0.8130<br>p > 0.9999	n = 7<br>AUC = 0.8367<br>CI = 0.6099-1.000<br>p = 0.0350
cell inject. (B)	n = 7<br>AUC = 0.5408<br>CI = 0.2050-0.8766<br>p = 0.7983	n = 7<br>AUC = 0.9592<br>CI = 0.8605-1.000<br>p = 0.0040	n = 7<br>AUC = 0.5918<br>CI = 0.2757-0.9079<br>p = 0.5653	n = 7<br>AUC = 0.5000<br>CI = 0.1870-0.8130<br>p > 0.9999	n = 7<br>AUC = 0.8367<br>CI = 0.5981-1.000<br>p = 0.0350
BDL (vehicle)	n = 9<br>AUC = 0.8642<br>CI = 0,6938-1.000<br>p = 0.0092	n = 9<br>AUC = 0.08557<br>CI = 0.7150-1.000<br>p = 0.0062	n = 7<br>AUC = 0.9898<br>CI = 0.9497-1.000<br>p = 0.0022	n = 9<br>AUC = 1.000<br>CI = 1.000-1.000<br>p = 0.0003	not done
BDL (MCC950)	n = 7<br>AUC = 0.9388<br>CI = 0.8067-1.000<br>p = 0.0060	n = 7<br>AUC = 1.000<br>CI = 1.000-1.000<br>p = 0.0017	n = 7<br>AUC = 0.9898<br>CI = 0.9497-1.000<br>p = 0.0022	n = 7<br>AUC = 0.8571<br>CI = 0.6381-1.000<br>p = 0.0253	not done

Table S3. Supplemental information to ROC curve analysis of figure 4. Analysis of various methods such as percentage of body weight change (%BW), burrowing activity (B), nesting behavior (N), a distress score (DSC) or fecal corticosterone metabolites concentration (FCM), when differentiating between animals before and after induction of a disease. The table presents the number of animals (n) analyzed, the area under the curve (AUC), the confidence interval (CI), and the p value describing the significant difference to a reference line, which indicates no discriminative power and thus has an AUC of 0.5.

	%BW	B	N	DSC	FCM
cholestasis (vehicle)	n = 9<br>AUC = 0.9218<br>CI = 0.8343-1.000<br>p = 0.0002	n = 9<br>AUC = 0.7716<br>CI = 0.5976-0.9456<br>p = 0.0159	n = 7<br>AUC = 0.8231<br>CI = 0.6586-0.9877<br>p = 0.0117	n = 9<br>AUC = 1.000<br>CI = 1.000-1.000<br>p < 0.0001	not done
cholestasis (MCC950)	n = 7<br>AUC = 0.9320<br>CI = 0.8327-1.000<br>p = 0.0008	n = 7<br>AUC = 0.9728<br>CI = 0.9129-1.000<br>p = 0.0002	n = 7<br>AUC = 0.8912<br>CI = 0.7240-1.000<br>p = 0.0023	n = 7<br>AUC = 1.000<br>CI = 1.000-1.000<br>p < 0.0001	not done
CCl4 intox. (vehicle)	increased	n = 3<br>AUC = 0.6111<br>CI = 0.2619-0.9603<br>p = 0.5791	n = 6<br>AUC = 0.8796<br>CI = 0.7437-1.000<br>p = 0.0063	n = 3<br>AUC = 0.5556<br>CI = 0.1838-0.9273<br>p = 0.7815	not done
CCl4 intox. (MCC950)	increased	n = 7<br>AUC = 0.8639<br>CI = 0.7200-1.000<br>p = 0.1762	n = 6<br>AUC = 0.9306<br>CI = 0.8216-1.000<br>p = 0.0019	n = 7<br>AUC = 0.5000<br>CI = 0.2487-0.7513<br>p > 0.9999	not done
cancer (vehicle)	n = 7<br>AUC = 0.7109<br>CI = 0.5109-0.9108<br>p = 0.1000	n = 7<br>AUC = 0.5272<br>CI = 0.2708-0.7836<br>p =0.8319	n = 7<br>AUC = 0.5306<br>CI = 0.2416-0.8197<br>p = 0.8113	n = 7<br>AUC = 0.5000<br>CI = 0.2487-0.7513<br>p > 0.9999	n = 7<br>AUC = 0.5034<br>CI = 0.2531-0.7537<br>p = 0.9788
cancer (CHC+Met)	n = 7<br>AUC = 0.7245<br>CI = 0.5299-0.9191<br>p = 0.0800	n = 7<br>AUC = 0.6190<br>CI = 0.4036-0.8345<br>p = 0.3532	n = 7<br>AUC = 0.6633<br>CI = 0.4417-0.8849<br>p = 0.2029	n = 7<br>AUC = 0.5714<br>CI = 0.3382-0.8047<br>p = 0.5775	n = 7<br>AUC = 0.5069<br>CI = 0.2786-0.7350<br>p = 0.9577
pancreatitis (vehicle)	n = 8<br>AUC = 0.9740<br>CI = 0.9272-1.000<br>p < 0.0001	n = 8<br>AUC = 0.8854<br>CI = 0.7678-1.000<br>p = 0.0013	n = 8<br>AUC = 0.7786<br>CI = 0.5826-0.9747<br>p = 0.0199	n = 8<br>AUC = 0.6042<br>CI = 0.3948-0.8135<br>p = 0.3841	n = 8<br>AUC = 0.7370<br>CI = 0.5676-0.9063<br>p = 0.0477
pancreatitis (miR-21 inhib.)	n = 8<br>AUC = 0.8568<br>CI = 0.7229-0.9906<br>p = 0.0029	n = 8<br>AUC = 0.8177<br>CI = 0.6503-0.9851<br>p = 0.0079	n = 8<br>AUC = 0.8880<br>CI = 0.7761-1.000<br>p = 0.0012	n = 8<br>AUC = 0.5208<br>CI = 0.2911-0.7506<br>p = 0.8618	n = 8<br>AUC = 0.6536<br>CI = 0.4491-0.8582<br>p = 0.1992

Table S4. Supplemental information to ROC curve analysis of figure 5. Analysis of various methods such as percentage of body weight change (%BW), burrowing activity (B), nesting behavior (N), a distress score (DSC) or fecal corticosterone metabolites concentration (FCM), when differentiating between animals before and at the early, middle or late stage of a disease. The table presents the number of animals (n) analyzed, the area under the curve (AUC), the confidence interval (CI), and the p value describing the significant difference to a reference line, which indicates no discriminative power and thus has an AUC of 0.5.

	BW	B	N	DSC	FCM
cholestasis early	n = 16<br>AUC = 0.8887<br>CI = 0.7626-1.000<br>p = 0.0002	n = 16<br>AUC = 0.7598<br>CI = 0.5913-0.9283<br>p = 0.0122	n = 14<br>AUC = 0.9617<br>CI = 0.8931-1.000<br>p < 0.0001	n = 16<br>AUC = 1.000<br>CI = 1.000-1.000<br>p < 0.0001	not done
cholestasis middle	n = 16<br>AUC = 0.9199<br>CI = 0.8215-1.000<br>p < 0.0001	n = 16<br>AUC = 0.7891<br>CI = 0.6147-0.9634<br>p =0.0053	n = 14<br>AUC = 0.8571<br>CI = 0.7048-1.000<br>p = 0.0013	n = 16<br>AUC = 1.000<br>CI = 1.000-1.000<br>p < 0.0001	not done
cholestasis late	n = 16<br>AUC = 0.9766<br>CI = 0.9322-1.000<br>p < 0.0001	n = 16<br>AUC = 0.9570<br>CI = 0.8915-1.000<br>p < 0.0001	n = 14<br>AUC = 0.8980<br>CI = 0.7774-1.000<br>p = 0.0003	n = 16<br>AUC = 1.000<br>CI = 1.000-1.000<br>p < 0.0001	not done
CCl4 intox. early	increased	n = 10<br>AUC = 0.5850<br>CI = 0.3277-0.8423<br>p = 0,5205	n = 12<br>AUC = 0.7257<br>CI = 0.5175-0.9339<br>p = 0.0606	n = 10<br>AUC = 0.5000<br>CI = 0.2407-0.7593<br>p > 0.9999	not done
CCl4 intox. middle	increased	n = 10<br>AUC = 0.6850<br>CI = 0.4422-0.9278<br>p = 0.1620	n = 12<br>AUC = 1.000<br>CI = 1.000-1.000<br>p < 0.0001	n = 10<br>AUC = 0.5500<br>CI = 0.2920-0.8080<br>p = 0.7055	not done
CCl4 intox. late	increased	n = 10<br>AUC = 0.6950<br>CI = 0.4565-0.9335<br>p = 0.1405	n = 12<br>AUC = 1.000<br>CI = 1.000-1.000<br>p < 0.0001	n = 10<br>AUC = 0.5000<br>CI = 0.2407-0.7593<br>p > 0.9999	not done
cancer early	n = 14<br>AUC = 0.5944<br>CI = 0.3768-0.8119<br>p = 0.3953	n = 14<br>AUC = 0.7883<br>CI = 0.6161-0.9604<br>p = 0.0094	n = 14<br>AUC = 0.6020<br>CI = 0.3885-0.8156<br>p = 0.3581	n = 14<br>AUC = 0.5357<br>CI = 0.3186-0.7528<br>p = 0.7477	n = 14<br>AUC = 0.5026<br>CI = 0.2761-0.7290<br>p = 0.9817
cancer middle	n = 14<br>AUC = 0.7398<br>CI = 0.5522-0.9274<br>p =0.0308	n = 14<br>AUC = 0.5051<br>CI = 0.2861-0.7241<br>p = 0.9634	n = 14<br>AUC = 0.5383<br>CI = 0.3203-0.7562<br>p = 0.7304	n = 14<br>AUC = 0.5357<br>CI = 0.3186-0.7528<br>p = 0.7477	n = 14<br>AUC = 0.5561<br>CI = 0.3308-0.7815<br>p = 0.6133
cancer late	n = 14<br>AUC = 0.7577<br>CI = 0.5700-0.9453<br>p = 0.0203	n = 14<br>AUC = 0.6582<br>CI = 0.4483-0.8680<br>p = 0.1543	n = 14<br>AUC = 0.5918<br>CI = 0.3788-0.8049<br>p =0.4082	n = 14<br>AUC = 0.5357<br>CI = 0.3186-0.7528<br>p = 0.7477	n = 14<br>AUC = 0.5255<br>CI = 0.3064-0.7446<br>p = 0.8183
pancreatitis early	n = 16<br>AUC = 0.8477<br>CI = 0.7099-0.9854<br>p = 0.0008	n = 16<br>AUC = 0.9102<br>CI = 0.8140-1.000<br>p = 0.0001	n = 16<br>AUC = 0.7109<br>CI = 0.5309-0.8909<br>p = 0.0418	n = 16<br>AUC = 0.5000<br>CI = 0.2969-0.7031<br>p > 0.9999	n = 16<br>AUC = 0.8359<br>CI = 0.6955-0.9764<br>p = 0.0012
pancreatitis middle	n = 16<br>AUC = 0.9395<br>CI = 0.8557-1.000<br>p < 0.0001	n = 16<br>AUC = 0.8008<br>CI = 0.6417-0.9598<br>p = 0.0037	n = 16<br>AUC = 0.9023<br>CI = 0.7901-1.000<br>p < 0.0001	n = 16<br>AUC = 0.5938<br>CI = 0.3942-0.7933<br>p = 0.3657	n = 16<br>AUC = 0.5547<br>CI = 0.3493-0.7600<br>p = 0.5977
pancreatitis late	n = 16<br>AUC = 0.9395<br>CI = 0.8589-1.000<br>p < 0.0001	n = 16<br>AUC = 0.8281<br>CI = 0.6775-0.9788<br>p = 0.0015	n = 16<br>AUC = 0.8789<br>CI = 0.7542-1.000<br>p = 0.0003	n = 16<br>AUC = 0.5938<br>CI = 0.3942-0.7933<br>p = 0.3657	n = 16<br>AUC = 0.7188<br>CI = 0.5296-0.9079<br>p = 0.0348
